# Childhood Arthritis and Rheumatology Research Alliance consensus clinical treatment plans for juvenile dermatomyositis with skin predominant disease

**DOI:** 10.1186/s12969-016-0134-0

**Published:** 2017-01-11

**Authors:** Susan Kim, Philip Kahn, Angela B. Robinson, Bianca Lang, Andrew Shulman, Edward. J. Oberle, Kenneth Schikler, Megan Lea Curran, Lilliana Barillas-Arias, Charles H. Spencer, Lisa G. Rider, Adam M. Huber

**Affiliations:** 1 Division of Pediatric Rheumatology, Benioff Children’s Hospital, University of California at San Francisco, 550 16th St, San Francisco, CA USA; 2 Division of Pediatric Rheumatology, New York University Langone Medical Center, 550 First Avenue, New York, NY USA; 3 Pediatric Rheumatology, Rainbow Babies and Children’s Hospital, 11100 Euclid Ave MS6008B, Cleveland, OH USA; 4 Department of Pediatrics, IWK Health Centre and Dalhousie University, 5980 University Ave, Halifax, NS Canada; 5 Pediatric Rheumatology, Children’s Hospital of Orange County, 1201 W La Veta Ave, Irvine, CA USA; 6 Department of Pediatrics, The Research Institute at Nationwide Children’s Hospital, 700 Children’s Dr, Columbus, OH USA; 7 Divisions of Adolescent Medicine and Pediatric Rheumatology, Department of Pediatrics, University of Louisville School of Medicine, 571 South Floyd St, Louisville, KY USA; 8 Department of Pediatrics, Northwestern University Feinberg School of Medicine, Chicago, Illinois; Division of Rheumatology, Ann and Robert H. Lurie Children’s Hospital of Chicago, 225 E Chicago Ave, Chicago, IL USA; 9 Department of Pediatrics, Rheumatology, Albany Medical Center, 43 New Scotland Ave, Albany, NY USA; 10 Environmental Autoimmunity Group, Clinical Research Branch, National Institute of Environmental Health Sciences, National Institutes of Health, 10 Center Drive, Bethesda, MD USA

**Keywords:** Dermatomyositis, Childhood Type, Therapeutics, Child, Adolescent, Amyopathic

## Abstract

**Background:**

Juvenile dermatomyositis (JDM) is the most common form of the idiopathic inflammatory myopathies in children. A subset of children have the rash of JDM without significant weakness, and the optimal treatments for these children are unknown. The goal of this study was to describe the development of consensus clinical treatment plans (CTPs) for children with JDM who have active skin rashes, without significant muscle involvement, referred to as skin predominant JDM in this manuscript.

**Methods:**

The Children’s Arthritis and Rheumatology Research Alliance (CARRA) is a North American consortium of pediatric rheumatology health care providers. CARRA members collaborated to determine consensus on typical treatments for JDM patients with skin findings without significant weakness, to develop CTPs for this subgroup of patients. We used a combination of Delphi surveys and nominal group consensus meetings to develop these CTPs.

**Results:**

Consensus was reached on patient characteristics and outcome assessment, and CTPs were developed and finalized for patients with skin predominant JDM. Treatment option A included hydroxychloroquine alone, Treatment option B included hydroxychloroquine and methotrexate, and Treatment option C included hydroxychloroquine, methotrexate and corticosteroids.

**Conclusions:**

Three CTPs were developed for use in children with skin predominant JDM, which reflect typical treatment approaches. These are not considered to be specific recommendations or standard of care. Using the CARRA network and prospective data collection, we will be able to apply statistical methods in the future to allow comparisons of JDM patients following these consensus treatment plans.

## Background

Juvenile Dermatomyositis (JDM) is the most common form of the idiopathic inflammatory myopathies in children but is relatively rare, affecting about 1–3 per 1 million children annually in the United States [1, 2]. It is a diffuse vasculopathy with inflammation in skin and muscle, typically associated with weakness and physical limitations.

Classic cutaneous manifestations which are pathognomonic for the diagnosis of JDM include Gottron’s papules and heliotrope rash. Malar rash and nailbed capillary changes are also frequently present [3]. JDM patients typically have proximal muscle weakness, but a subset of patients present with skin disease without any significant weakness or muscle inflammation [4, 5].

General designations for this subset of JDM patients have included: amyopathic dermatomyositis (DM), clinically amyopathic DM, DM *sine* myositis, and hypomyopathic DM, among others [5–9]. These generally have intended to describe the subset of DM patients with typical skin disease of DM but without clinically significant weakness. For this publication, we will use the term *skin predominant JDM* to describe the patient subtype under consideration.

The epidemiology, treatment and outcomes of skin predominant JDM are not well studied. The largest series of clinically amyopathic JDM patients published included 68 cases, of which about 25% progressed to classical JDM [10]. Few patients with clinically amyopathic JDM in this series developed disease-related complications: only 4% developed calcinosis and no patients developed vasculopathy, interstitial lung disease or malignancy [10].

The best treatments for JDM and skin predominant JDM are not known, and treatments used are extremely variable [11]. Proposed therapy of skin predominant JDM includes recommendations for topical and systemic therapies, without trial data or case experiences to support these approaches [12].

Presently available retrospective studies in JDM are limited by small sample sizes, lack of blinding, and lack of generalizability. Efforts to conduct a traditional randomized controlled trial to conclusively evaluate the best treatment of skin predominant JDM are associated with many challenges. First, the rarity of skin predominant JDM, which is a subset of an already uncommon condition, limits its successful study due to the inability to accrue sufficient numbers of patients from a reasonable number of sites, over a practical time period. In addition, there are significant cost and logistic issues involved in conducting traditional clinical trials in this uncommon condition.

To overcome these obstacles, the Children’s Arthritis and Rheumatology Research Alliance (CARRA) has worked to develop consensus treatment plans (CTPs) to study treatments in patients with rare rheumatic diseases like JDM [13–15] and other pediatric inflammatory conditions [16–18] to allow for pragmatic studies of treatments and outcomes. These have been termed consensus clinical treatment plans, as they have been developed by CARRA members, through consensus methods. These CTPs are meant to represent typical, commonly accepted treatment approaches used by pediatric rheumatologists to treat these illnesses. These commonly accepted treatment approaches are generally based on anecdotal experience and have not been studied in the context of any formal clinical trials.

The intent of the CTP is that each treating clinician will be able to choose and follow a plan for the care of their individual patient, which is similar to their typical treatment approach. This will allow for increased standardization of treatment approaches between institution review board (IRB) approved CARRA centers and clinicians, and facilitate the prospective collection of data through CARRA’s detailed registry, including: patient demographics, clinical and lab characteristics, medication-related adverse events, as well as response to treatments and outcomes. Using patient characteristics and outcomes that will be collected prospectively, innovative statistical methods can be used to account for the selection biases of non-random assignment to CTP treatment groups, to estimate treatment effects comparable to those that might be obtained with traditional randomized controlled trials [19–23].

Previously, a CTP for children presenting with moderate JDM was published by CARRA [13, 14], and a pilot study using that CTP was recently completed and undergoing analysis. A second CTP was developed and recently published for *skin resistant* JDM, characterized by persistent skin rash despite resolution of muscle involvement [15]. The goal of the present study is to describe the development of a third set of JDM CTPs for *skin predominant* JDM, which applies to the distinct subset of JDM patients, whose presentation is characterized by classic skin rash without significant muscle involvement.

## Methods

CARRA is a North American organization, made up of pediatric rheumatologists and other medical professionals, interested in advancing research in pediatric rheumatic conditions. It is comprised of more than 400 individuals from more than 110 centers, which includes the majority of pediatric rheumatologists in North America. CARRA’s mission is to “conduct collaborative research to prevent, treat and cure pediatric rheumatic diseases”.

This CTP was developed over several years of collective work and consensus building, with our CARRA collaborators, to represent typical care provided in the pediatric rheumatology community. At each meeting, results from previous surveys, consensus meetings, as well as relevant literature were reviewed and presented to participants. Surveys were sent to the complete CARRA membership and members were asked to complete the survey if they treated patients with JDM and had sufficient expertise.

1. CARRA Annual Meeting, 2011—Miami, FL. During this meeting, approximately 36 members of the CARRA JDM Committee discussed the clinically relevant JDM phenotypes for which additional CTPs should be developed. It was agreed that “skin disease” in JDM was important and specifically patients presenting with rash but no clinical weakness were considered to be a clinically relevant subtype of JDM (so called “amyopathic and hypomyopathic” JDM), which would include *newly* diagnosed JDM patients. It was also agreed that patients with “resistant skin disease” (i.e. patients with typical JDM, who had persistent skin disease despite resolution of muscle involvement following treatment) were a distinct JDM subtype. It was agreed that these subgroups should be considered separately [15].
  There was general discussion regarding patient characteristics of “amyopathic and hypomyopathic” JDM and at this meeting, and it was decided that the preferred descriptor for this subtype of JDM patient would be “skin predominant JDM”. In particular, since some cutaneous features of JDM are considered pathognomonic, initiating treatment for patients in this CTP with at least 6 weeks of skin findings was agreed upon.
2. CARRA Annual Meeting, 2012—Las Vegas, NV. A smaller core work group of 7 CARRA members from the JDM Committee met to begin developing components of the CTP, including the definition of skin predominant JDM, inclusion and exclusion criteria and a broad range of reasonable treatment regimens, and four candidate treatment arms were agreed upon. The initial candidate treatment arms included 1) hydroxychloroquine alone, 2) hydroxychloroquine and methotrexate, 3) hydroxychloroquine, methotrexate and intravenous immunoglobulin, and 4) hydroxychloroquine, methotrexate, intravenous immunoglobulin and corticosteroids.
3. Delphi Survey, Spring 2013— An electronic survey was sent to all CARRA members (n = 400), to present the consensus results from the 2012 meeting for clarification regarding issues that had not been satisfactorily addressed in the meetings. These issues included whether skin predominant JDM was broadly considered to be an important clinical issue, and if members would consider using a CTP for the treatment of this JDM subtype. Complete responses were received from 97 CARRA members (73 pediatric rheumatologists, 7 internal medicine/pediatric rheumatologists and 17 pediatric rheumatology fellows). Of these, 31% had 0–4 years, 26% had 5–10 years and 43% had >10 years of experience caring for patients with JDM. More than 82% of respondents considered themselves to be moderately or very experienced in the care of JDM. The majority of respondents agreed that skin predominant JDM was an important clinical issue, and would consider using a CTP to treat this JDM subtype (92% and 98%, respectively). In addition, 91% of respondents agreed that rash for ≥ 6 weeks was sufficient to include a patient in this CTP.
4. CARRA Annual Meeting, 2013—Chicago, IL. Building on the results from the consensus meeting from the previous year and the Delphi survey results, a core group of 21 members of the CARRA JDM Committee met. Nominal group methods [24] were used to come to consensus, which was set at ≥75%. Issues addressed included clarification of patient inclusion and exclusion criteria, minimum evaluation for inclusion into these CTPs, interval of monitoring and data collection, medications to be included, the use of topical therapies, and the role of additional diagnostic testing, including imaging and biopsies. After additional review and discussion of the candidate treatment arms, consensus was reached to exclude the treatment option that included IVIG, decreasing the number of treatment arms to three. During the course of this meeting, concern was expressed regarding the rarity of skin predominant JDM, and it was suggested that perhaps pediatric dermatologists were independently seeing and managing this JDM subtype.
5. January 2014— Additional expertise was requested and obtained from Pediatric Dermatology (PeDRA) members in 2014. An electronic survey was sent out to all PeDRA members (n = 146) to better understand the degree to which they were managing and treating skin predominant JDM. Forty PeDRA members responded to the survey: 39 (98%) of the respondents practiced in an academic setting. Most had been in practice more than 5 years: 7 (18.0%) for 6–9 years and 17 (43.6%) for >10 years. All respondents agreed that better treatment and understanding of this JDM subtype was important. Thirty five (90%) reported that they refer all JDM patients to a pediatric rheumatologist, and 30 (77%) estimated that they see 1–5 amyopathic JDM patients within the past 5 years.
6. CARRA Annual Meeting, 2014—Orlando, FL. Twelve members of the CARRA JDM Committee met to further develop the CTPs and formally review the PeDRA survey results. Nominal group methods [24] were used to come to consensus, which was set at ≥75%. Further decisions regarding treatment arms, medication dosing, use of topical therapies and outcome assessment were made.
7. CARRA Annual Meeting, 2015—Austin, TX. Ten members of the CARRA JDM Committee met to finalize the proposed CTP. In addition, 2 PeDRA members and one patient/parent representative from CureJM participated in the meeting. The proposed CTP was presented and using nominal group methods [24] the complete CTP proposal was finalized.
  A number of modifications were considered in this meeting. Clarification regarding the age of JDM patients and duration of symptoms needed were made. Inclusion criteria were discussed in detail, especially the definition of normal strength and how it is determined by clinicians in light of the challenges of strength testing in young children. The three treatment arms were finalized, and all participants agreed that topical therapies would be explicitly monitored with this CTP. All respondents agreed that presently available and validated tools should be used to assess skin activity. Final consensus was reached regarding recommendations on steroid dose.
  It was agreed that patients would be withdrawn from this CTP if they developed weakness during the course of treatment or required additional systemic therapy. We reviewed and summarized our work to date in order to develop CTPs which reflected CARRA consensus on typical treatments for JDM patients with skin predominant disease.

## Results

The CTPs presented in this manuscript are intended for patients with skin predominant JDM. CARRA members reached consensus on the clinical characteristics of patients to be included in this subgroup of JDM (Table 1). These patients are defined as children with cutaneous manifestations of JDM skin disease for at least 6 weeks, without any weakness detected by the patient/parent or clinician. All patients included in this CTP require one of the hallmark rashes of JDM, namely, Gottron’s papules or heliotrope rash, with or without other cutaneous manifestations (e.g. Shawl sign, V-sign, malar rash, periungal erythema, vasculopathic changes in the nail bed capillaries, etc.).

**Table 1:** Patient Characteristics for Skin Predominant Juvenile Dermatomyositis (JDM)

Patients Inclusion Characteristics
1. Typical rash consistent with JDM for ≥6 weeks.
a. Should include Gottron’s papules or heliotrope rash
b. May have mild calcinosis and nailbed capillary abnormalities
2. No functional limitations or weakness
a. Based on history or CHAQ
b. As determined by treating physician based on strength assessment
3. Muscle enzymes ≤ 1.2 times upper limit of normal
a. Except if attributed to another process, such as exercise or drug therapy
Patients Exclusion Characteristics
1. Significant systemic involvement, including parenchymal lung disease, dysphagia, aspiration, intestinal vasculitis, myocarditis
2. Significant calcinosis, as determined by treating clinician
3. Ulcerative skin disease
4. Lipodystrophy
5. Pregnancy

Abbreviations: *JDM* juvenile dermatomyositis; *CHAQ* Childhood Health Assessment Questionnaire

Patients may have mild calcinosis, determined by the judgement of the treating clinician, but should not have lipodystrophy or skin ulceration, since it was agreed that patients with these findings have greater disease severity beyond the context of this CTP. In addition, patients should not have systemic involvement or findings of other major organ involvement, including parenchymal lung disease, dysphagia, aspiration, intestinal vasculitis, or myocarditis, as assessed by the treating clinician.

Medication therapy in each of the CTPs is summarized in Table 2. All treatment plans include hydroxychloroquine at 5mg/kg/day (maximum 400mg), with this as monotherapy in Treatment option A. Treatment option B includes the additional use of methotrexate, administered by the parenteral route (preferred), at a dose of 15 mg/m^2^ or 1 mg/kg (maximum 40 mg) once weekly. Treatment option C includes oral corticosteroids (prednisone at 1–2 mg/kg/day, maximum 60 mg) in conjunction with weekly methotrexate, and daily oral hydroxychloroquine in the same doses as treatment Plan B.

**Table 2:** Consensus clinical treatment plans for Patients with Skin Predominant Juvenile Dermatomyositis*

All patients should be asked to use optimal sun protection, including regular use of broad spectrum sunscreen or sun block of SPF ≥ 30
All other topical therapies (steroids, calcineurin inhibitors, etc.) should be recorded
Treatment A
Hydroxychloroquine: 5mg/kg/day, maximum 400mg
Treatment B
Methotrexate subcutaneous, unless only oral administration is possible −15 mg/m^2^ or 1 mg/kg (maximum 40 mg) once/week Hydroxychloroquine: 5mg/kg/day, maximum 400mg
Treatment C
Prednisone −1–2 mg/kg/day (maximum 60 mg) Methotrexate subcutaneous, unless only oral administration is possible −15 mg/m^2^ or 1 mg/kg (maximum 40 mg) once/week Hydroxychloroquine: 5mg/kg/day, maximum 400mg

*Patients who develop weakness defined as need for additional disease-modifying antirheumatic drugs, or any of the following: decline in the MDAAT, MD Global, Extramuscular Disease Activity by ≥ 2cm or worsening of muscle enzymes by ≥20%. would be withdrawn from this CTP but may then be eligible to enter the CTPs for moderate JDM (13,14)

In all CTPs, appropriate sun avoidance and maximization of sunscreen use, including broad spectrum products with SPF ≥ 30, were recommended for all patients, with specific recommendations according to the treating clinician. It was agreed that details regarding topical therapies, which would include any topical steroids and topical calcineurin inhibitors, including various formulations and potencies, would be collected and recorded in a detailed manner in the CARRA registry.

Since these CTPs deal with skin disease as the major manifestation of JDM and resolution of skin features is the primary outcome measure, there was extensive discussion about how skin disease would be assessed. Use of presently available assessment tools [25–29] was weighed against the development of a novel assessment tool more applicable to these specific CTPs. It was decided that the cutaneous disease activity visual analogue scale (VAS) from the Myositis Disease Activity Assessment Tool (MDAAT) would serve as the primary outcome [25, 30]. It was agreed that the development of a novel tool was outside the scope of this current work. This was a consensus decision supported by 91% of respondents in the initial Delphi survey and 100% of participants during the 2015 CARRA meeting.

Baseline investigations and data collection at follow-up visits were common to all 3 CTPs (Table 3). It was agreed that all patients would have basic investigations at enrollment and at follow-up visits, suggested at a 3 to 4 month interval, to minimize the burden on busy clinicians. In addition, centers would have the option of collecting additional data including autoantibody studies and either the PRINTO or IMACS core set activity measures [25, 31, 32] at baseline and at follow-up.

**Table 3:** Initial and Follow Up Assessments for Skin Predominant Juvenile Dermatomyositis Patients

Initial
A. Basic Assessment
a. Cutaneous Disease Activity Visual Analogue Scale from the Myositis Disease Activity Assessment Tool (MDAAT)
b. Physician global assessment of disease activity (10-cm VAS)
c. Patient/Parent global assessment of disease activity (10-cm VAS)
d. Physician Extramuscular disease activity (10-cm VAS)
e. CHAQ
f. Strength Testing: CMAS or Manual muscle testing
g. Documentation of muscle involvement if performed (e.g. MRI, EMG, biopsy results)
h. Muscle enzymes (preferably several among serum levels of ALT, AST, LDH, CK, aldolase)
i. Baseline laboratory testing (include complete blood cell count, immunoglobulins)
j. Nailfold capillaroscopy (using hand-held magnifier, ophthalmoscope, or microscope)
B. Expanded assessment (Basic plus items below)
a. ANA and other autoantibodies (myositis-specific and myositis-associated)
b. Full PRINTO core set activity measures, including Disease Activity Score and Health-Related Quality of Life* [32]
Follow-up
A. Basic Assessment
a. Cutaneous Disease Activity Visual Analogue Scale from the Myositis Disease Activity Assessment Tool (MDAAT)
b. Physician global assessment of disease activity (10-cm VAS)
c. Patient/Parent global assessment of disease activity (10-cm VAS)
d. Physician Extramuscular disease activity (10-cm VAS)
e. CHAQ
f. Strength Testing: CMAS or Manual muscle testing
g. Muscle enzymes (preferably several of ALT, AST, LDH, CK, aldolase)
h. Nailfold capillaroscopy (using hand-held magnifier, ophthalmoscope, or microscope)
B. Expanded Assessment (Basic plus items below)
a. Full PRINTO core set activity measures [32]

* Note the basic assessment includes the International Myositis Assessment and Clinical Studies (IMACS) Group core set measures for JDM [25]

Abbreviations: *MDAAT* Myositis Disease Activity Assessment Tool; *VAS* Visual Analog Scale; *CHAQ* Childhood Health Assessment Questionnaire; *CMAS* Childhood Myositis Assessment Scale; *MRI* magnetic resonance imaging; *EMG* Electromyography; *ALT* alanine aminotransferase; *AST* aspartate aminotransferase; *LDH* lactate dehydrogenase; *CK* creatine kinase; *ANA* antinuclear antibody; *PRINTO* Pediatric Rheumatology International Trials Organization

It was agreed that treatment failure was defined as the addition of any additional disease-modifying antirheumatic drugs to any of the three treatment options, or any of the following: decline in the MDAAT, MD Global, Extramuscular Disease Activity by ≥ 2 cm or worsening of muscle enzymes by ≥20%. These patients would be withdrawn from the CTP.

## Discussion

We present a set of consensus clinical treatment plans (CTPs) for children with JDM who have typical skin rashes but no significant muscle weakness, which we call skin predominant JDM.

Skin disease as a component of JDM is important since it reflects disease activity and may contribute to morbidity, including physical disfigurement, calcinosis and lipodystophy. Skin disease is a manifestation of active vasculopathy in JDM that is important to monitor and treat [33, 34]. It has been reported that early severe skin disease activity in JDM may predict cardiac dysfunction better than muscle disease [35], and persistently active skin disease at 3 and 6 months after diagnosis is predictive of continued active disease [36]. These data support the importance of adequate treatment of JDM-related skin disease, since it may predict worse outcomes [33–36]. There is limited knowledge of patients with skin predominant JDM and optimal treatment approaches, and studying this rare condition using traditional clinical trials is not possible.

These CTPs are meant to represent typical, reasonable treatment approaches taken by pediatric rheumatologists. They are not standard of care, and do not replace clinical judgment or decision making between the treating physician and patient. The intent of these CTPs are to provide clinicians with reasonable medication options for treating patients with skin predominant JDM, in hopes of diminishing differences in treatment approaches between clinicians across multiple CARRA centers. This in turn will allow for improved prospective data collection of patient responses to treatments through the CARRA registry. Using statistical methods [22, 23], this prospective and uniform data collection will allow us to compare differences in patient outcomes in the three different CTP options, which may estimate treatment effects similar to those obtained with traditional randomized controlled trials.

There are important limitations to consider with this work. We chose to include patients with classic skin findings of JDM for a minimum of 6 weeks. Though most case definitions of this patient subtype suggest 6 months before formal classification [10], we note that in clinical practice, treatment is usually initiated in a JDM patient based on clinical indication, rather than an exact duration of symptoms. Therefore we felt that inclusion of these early patients for this CTP was reasonable and reflected CARRA member practice patterns. In addition, we are aware that nearly 25% of patients with skin predominant JDM progress to classic JDM with weakness, over time. Thus, we included criteria for treatment failure, and guidelines for withdrawal from this CTP, expecting that some patients would evolve to classic JDM with weakness over time. We hypothesize that early introduction of systemic treatment in skin predominant JDM may delay or prevent the development of weakness, so formal study of this is planned in the context of this work.

We involved a large number of pediatric rheumatologists in this consensus process, over the course of several years. Responses to online surveys from CARRA and PeDRA members were about 24 and 27%, respectively, which can be considered a low response rate. We found that survey responses pertaining to this topic are lower compared to earlier surveys from CARRA [13, 14, 16, 17], however, it should be noted that our present survey response rates are comparable to current reported online survey response rates [37, 38]. The surveys were sent to the full CARRA and PeDRA membership, and not a specific subset of JDM experts, but it is reasonable to postulate that clinicians with more exposure and expertise to JDM were more likely to respond to the surveys. We postulate that survey respondents were likely clinicians who have the most interest and expertise in JDM, so it is possible that these findings are not representative of all CARRA and PeDRA members. In this regard, additional Delphi survey of the full participating group could have been administered after finalizing the CTPs; however, given the general response to the earlier Delphi surveys, this was not performed. A previous CARRA survey, which collected information on medications used by pediatric rheumatologists in North America to treat skin predominant JDM, reported medications combinations which overlap considerably with the CTPs developed here [11]. Therefore, we are confident our CTPS can be used broadly in clinical practice and for larger scale, future study.

Topical therapies (sunscreens, steroids, calcineurin inhibitors, etc.) are not explicitly included among the separate treatment arms. The focus on systemic therapy in this CTP, reflects consensus among the participating pediatric rheumatologists, who generally treat these patients with systemic therapy, rather than topical therapies alone. In addition, we recognized that the power to detect a difference between the CTP options would be further diminished if various combinations of topical therapies were included. However, since we cannot discount the role of topical therapies, which are used broadly in the treatment of skin predominant JDM, we plan to keep a strict record the various topical therapies used. We will also account for topical therapies in the final analysis, to assess if patients treated with various topical therapies (e.g., topical corticosteroids and calcineurin inhibitors at varying formulations and potencies) are associated with a difference in outcome.

We note that we have not included all the treatment options available, including early use of intravenous immunoglobulin therapy. Based on patient and clinician preferences, these CTPs may not be applicable to all patients with skin predominant JDM. These CTPs include systemic therapies, and are less immunosuppressive compared to previously published CTPs developed for patients with moderate weakness or ongoing rash, which included more potent medications including, high dose oral corticosteroids, pulse corticosteroids and intravenous gammaglobulin [13–15].

## Conclusions

In conclusion, we present CTPs which are complementary to other treatment plans previously developed by the various disease-specific committees of CARRA [13–18]. It is hoped that these CTPs can be used prospectively to improve the understanding of the best treatment approaches for this skin predominant subgroup of JDM patients and improve outcomes.

## Abbreviations

ANA: Antinuclear antibody
ALT: Alanine aminotransferase
AST: Aspartate aminotransferase
CARRA: Childhood Arthritis and Rheumatology Research Alliance
CHAQ: Childhood Health Assessment Questionnaire
CK: Creatine kinase
CMAS: Childhood Myositis Assessment Scale
CTP: Clinical Treatment Plan
EMG: Electromyography
JDM: Juvenile Dermatomyositis
LDH: Lactate dehydrogenase
MDAAT: Myositis Disease Activity Assessment Tool
MRI: Magnetic resonance imaging
PRINTO: Pediatric Rheumatology International Trials Organization
VAS: Visual Analog Scale
